# Matrine: A Promising Natural Product With Various Pharmacological Activities

**DOI:** 10.3389/fphar.2020.00588

**Published:** 2020-05-07

**Authors:** Hong Zhang, Linlin Chen, Xipeng Sun, Quanjun Yang, Lili Wan, Cheng Guo

**Affiliations:** ^1^Department of Pharmacy, Shanghai Sixth People's Hospital, Shanghai Jiao Tong University, Shanghai, China; ^2^School of Medicine, Shanghai Jiao Tong University, Shanghai, China

**Keywords:** matrine, cancer, inflammation, apoptosis, autophagy, cell cycle, natural product

## Abstract

Matrine is an alkaloid isolated from the traditional Chinese medicine *Sophora flavescens* Aiton. At present, a large number of studies have proved that matrine has an anticancer effect can inhibit cancer cell proliferation, arrest cell cycle, induce apoptosis, and inhibit cancer cell metastasis. It also has the effect of reversing anticancer drug resistance and reducing the toxicity of anticancer drugs. In addition, studies have reported that matrine has a therapeutic effect on Alzheimer's syndrome, encephalomyelitis, asthma, myocardial ischemia, rheumatoid arthritis, osteoporosis, and the like, and its mechanism is mainly related to the inhibition of inflammatory response and apoptosis. Its treatable disease spectrum spans multiple systems such as the nervous system, circulatory system, and immune system. The antidisease effect and mechanism of matrine are diverse, so it has high research value. This review summarizes recent studies on the pharmacological mechanism of matrine, with a view to providing reference for subsequent research.

## Introduction

Traditional Chinese medicine *Kushen* is the dry root of the leguminous plant *Sophora flavescens* Aiton, which has a long history of medicinal use in China. It is commonly used in the clinical treatment of traditional Chinese medicine for dysentery, eczema and pruritus. Compound Kushen Injection is a common dosage form of *Kushen* for clinical application, and the main component of Compound Kushen Injection is matrine. At present, Compound Kushen Injection has been put into clinical application in the adjuvant treatment of lung cancer (Wang et al., 2016), breast cancer (Ao et al., 2019), esophageal cancer (Zhang et al., 2018a), gastric cancer (Zhang et al., 2018b), colon cancer (Yu et al., 2017; Yang et al., 2018), liver cancer (Ma X. et al., 2016), and pancreatic cancer (Zhang et al., 2017). Compound Kushen injection is also used to relieve cancer-related pain (Guo et al., 2015). Matrine (molecular formula: C_15_H_24_N_2_O, molecular weight: 248.36 g/mol), a tetracyclo-quinolizindine alkaloid, is the main bioactive compound in *Kushen*, and more than 1 g of matrine can be extracted from 10 kg of *Kushen* (Lai et al., 2003; Liu X. J. et al., 2010). With the deepening of modern pharmacological research, the medicinal value of matrine has been further developed. At present, the basic researches on the antitumor and antiinflammatory effects of matrine are in a large volume, indicating that matrine has various pharmacological activities and potential for clinical application. In addition, matrine has a good prospect as a one-component drug in clinical practice, and single-component drugs have certain advantages over traditional Chinese medicine injections in quality control. In this paper, we summarized the pharmacological effects and mechanisms of matrine in order to provide reference for the follow-up study. Compared with the previous review of matrine (Rashid et al., 2019; Li et al., 2020), this paper makes comprehensive supplements of the pharmacological action and molecular mechanism of matrine.

## Anticancer Activity

The antitumor activity of matrine is mainly manifested in inhibiting the proliferation of cancer cells, blocking cell cycle, inducing apoptosis and inhibiting the metastasis of cancer cells. At the same time, matrine can reverse the drug resistance of anticancer drugs and reduce the toxicity of anticancer drugs. The anticancer spectrum of matrine is very wide, and it can inhibit many kinds of cancer cells. The anticancer effect and mechanism of matrine are discussed in the following sections sorted by cancer types.

### Lung Cancer

Lung cancer has the largest number of deaths among all cancers, and the 1-year survival rate of advanced patients is very low. There is always a great need for treatment in lung cancer (Blandin Knight et al., 2017). Matrine has a strong inhibitory effect on lung cancer cells. Matrine can block the cell cycle of lung cancer A549 cells in G1/G0 phase, upregulate the expression of microRNA (miR)-126, and then downregulate the expression of miR-126 target gene vascular endothelial growth factor (VEGF) and induce apoptosis (An et al., 2016). Matrine can also upregulate the expression of p53 and p21 and downregulate the expression levels of proliferating cell nuclear antigen (PCNA) and eukaryotic initiation factor 4E (eIF4E) to inhibit proliferation and migration (Lu et al., 2017). Matrine induces apoptosis in lung cancer cells, and also downregulates the expression of inhibitor of apoptosis protein (IAP) (Niu et al., 2014) and regulates the protein kinase B/glycogen synthase kinase-3β (AKT/GSK-3β) signaling pathway by regulating phosphatidylinositol 3-kinase (PI3K)/AKT/mammalian rapamycin target protein (mTOR) signaling pathway (Xie et al., 2018). For A549, NCI-H358 cells, matrine activates the p38 pathway by inducing reactive oxygen species (ROS) production, leading to caspase-dependent apoptosis, and inhibition of the p38 pathway by SB202190 partially prevents matrine-induced apoptosis (Tan et al., 2013). Matrine can also inhibit the proliferation and migration of lung cancer LA795 cells by regulating transmembrane protein 16A (TMEM16A), and inhibit the tumor growth of LA795 transplanted tumor mice (Guo et al., 2018a). Epithelial-mesenchymal transition (EMT) is closely related to tumor metastasis. Matrine can inhibit EMT and inhibit metastasis in nonsmall cell lung cancer by inhibiting the expression of paired box 2 (PAX2) (Yang J. et al., 2017). In the aspect of antilung cancer resistance, matrine can reverse the cisplatin-resistant lung cancer cells against apoptosis by regulating the β-catenin/survivin signaling pathway (Wang et al., 2015a). The development of epidermal growth factor receptor (EGFR) inhibitors is one of the difficulties in the treatment of lung cancer with EGFR mutation. Matrine treatment can reduce the expression of IL6, inhibit the activation of Janus tyrosine kinase/signal transducer and activator of transcription 3 (JAK1/STAT3) signaling pathway, decrease the expression of B-cell lymphoma-2 (Bcl2), inhibit cell growth, induce apoptosis, and enhance the inhibitory effect of afatinib on H1975 cells (Chen et al., 2017).

### Breast Cancer

Shao et al. (2013) reported that matrine can inhibit the proliferation of breast cancer MCF7, BT-474, and MDA-MB-231 cells, which may be related to the inhibition of inhibitory κ B kinase β (IKKβ) regulation of nuclear factor κ B (NF-κB) signaling pathway. Matrine can induce endoplasmic reticulum stress in MCF-7 cells, downregulate the expression of hexokinase II, inhibit energy metabolism, promote apoptosis (Xiao et al., 2017), and reverse the drug resistance of MCF-7/ADR cells. Adriamycin (ADR) accumulates in cells and induces apoptosis in MCF-7/ADR cells by modulating the PI3K/AKT signaling pathway (Zhou B. G. et al., 2018). Matrine can also regulate Wnt/β-catenin signaling pathway, inhibit the expression of VEGF, thereby inhibiting the proliferation of breast cancer 4T1, MCF-7 cells, inducing apoptosis, and inhibiting tumor growth in 4T1 tumor-bearing mice (Xiao et al., 2018).

### Liver Cancer

Matrine can induce mitochondrial dysfunction in HepG2 cells, cause oxidative stress in cells, destroy cell energy metabolism, initiate endogenous apoptosis by regulating Mammalian STE20-like protein kinase 1/c-Jun NH2-terminal kinase (MST1/JNK) signaling pathway (Cao et al., 2019), and also inhibit mitosis through PINK1/PARKIN pathway, then promote apoptosis (Wei R. et al., 2018). Matrine is also capable of inducing caspase-independent programmed cell death *via* Bid-mediated AIF translocation (Zhou et al., 2014). Matrine can also induce autophagy in HepG2 cells and MHCC97L cells (Zhang et al., 2010; Yang and Yao, 2015). In hepatocellular carcinoma HepG2 cells, AMP-activated protein kinase (AMPK) signaling inhibits p53 and inhibits autophagy. After AMPK is inhibited, autophagy is converted to apoptosis (Xie et al., 2015). In addition, matrine has a proliferation inhibitory effect on cisplatin-purified liver cancer SMMC-7721 stem cell-like SMMC-7721-sphere cells (Wang H. et al., 2018). In addition, matrine can inhibit the migration and invasion of hepatoma cells by EMT *via* the Phosphatase and tensin homology deleted on chromosome ten (PTEN)/AKT pathway (Wang Z. et al., 2018). Matrine combined with resveratrol can better inhibit the proliferation of hepatoma cells and induce cell cycle arrest and endogenous apoptosis (Ou et al., 2014). When matrine combined with sorafenib, apoptosis of hepatocarcinoma cells can be induced by inhibiting miR-21 and upregulating PTEN expression (Lin et al., 2014).

### Cholangiocarcinoma/Gallbladder Carcinoma

Matrine can induce choline cancer cell necrosis by increasing ROS production *via* the receptor-interacting protein 3/mixed lineage kinase domain like protein (Rip3/MLKL) pathway (Xu et al., 2017), and can also induce mitochondria-associated endogenous apoptosis in cholangiocarcinoma cells *via* the JAK2/STAT3 pathway (Yang et al., 2015). For gallbladder cancer cells, matrine can also inhibit proliferation and induce cell cycle arrest and apoptosis (Zhang et al., 2012).

### Pancreatic Cancer

Pancreatic cancer is the lowest 5-year survival rate of all solid tumors and is expected to be the second leading cause of cancer-related deaths in the United States by 2030 (Moffat and Epstein, 2019). Matrine can inhibit the proliferation and migration of pancreatic cancer Panc-1 cells, induce ROS production, and induce apoptosis, which is related to ROS/NF-κB/matrix metalloproteinase (MMP) pathway (Huang and Xin, 2018). Matrine can also inhibit the proliferation of KRAS-mutated pancreatic cancer MIAPACA2 and 8988T cells, inhibit autophagy by downregulating STAT3, and inhibit mitochondrial energy production (Cho et al., 2018). Ma Y. et al. (2015) reported that matrine downregulates the expression of MT1-MMP *via* Wnt signaling pathway and inhibits pancreatic cancer cell migration and invasion. Matrine can inhibit the expression of PCNA and induce apoptosis in BxPC-3 and Panc-1 cells, and has no significant effect on human normal liver HL-7702 cells at the same dose. It can inhibit the growth of tumor xenograft tumors *in vivo* (Liu T. et al., 2010).

### Gastric Cancer

Matrine can inhibit the proliferation and migration of gastric cancer SGC7901 cells by PI3K/AKT/uPA pathway (Peng et al., 2016). Matrine acts on gastric cancer SGC7901 cells, and miRNA screening revealed increased levels of eight miRNAs in the cell cycle pathway of target gene aggregation, while levels of 14 miRNAs in target mitogen-activated protein kinase (MAPK) signaling pathways were reduced (Li H. et al., 2014). Matrine can regulate the structure and subcellular distribution of vasodilator-stimulated phosphoprotein (VASP) in gastric cancer BGC823 cells, thereby inhibiting the adhesion and migration of cancer cells (Zhang et al., 2013). It has been reported that matrine can induce autophagy in gastric cancer SGC7901 cells, and at the same time, it can block the process of autophagy degradation by impairing the activity of lysosomal proteases, thus inducing death (Wang et al., 2013). However, studies have reported that matrine also induces protective autophagy, in which process matrine treatment does not directly inhibit the expression of AKT and its downstream effector mTOR and phosphorylation of p70 ribosomal protein S6 kinase (p70S6K), and inhibition of autophagy can enhance the killing of gastric cancer cells by matrine (Li et al., 2013).

### Colon Cancer

Matrine can induce cell cycle arrest in G1/G0 phase and induce apoptosis in human colorectal cancer cell lines LS174T, Caco-2, SW1116, and Rko. Compared with oxaliplatin, matrine The LS174T nude mouse xenograft model has less influence on physical strength and body weight (Gu et al., 2018). Matrine can inhibit tumor growth in rats with colorectal cancer model, which is associated with inhibition of high mobility group protein box 1 (HMGB1) signaling pathway (Fan et al., 2018). For both LoVo cells and HT29 cells, matrine can also induce apoptosis (Chang et al., 2013; Zhang et al., 2014).

### Prostate Cancer

Li et al. (2018) used the Hiseq 2500 high-throughput sequencing platform to screen the proliferation inhibition mechanism of matrine on prostate cancer PC-3 and DU145 cells. The results showed that matrine inhibited cell proliferation, migration, and invasion through Forkhead box protein O (FoxO) and PI3K/AKT signaling pathways. Induction of apoptosis. Studies have shown that matrine upregulates gadd45b expression *via* p38/JNK, ROS/gadd45b/p38 pathway, inhibits proliferation and migration of prostate cancer DU145, PC3 cells, and induces apoptosis (Huang et al., 2018), which is also associated with NF-κB pathway (Li Q. et al., 2016). Matrine can inhibit the proteasome CTLIKE activity by activating the unfolded protein response/endoplasmic reticulum (UPR/ER) pathway, arresting the cell cycle in the G0/G1 phase, inducing apoptosis of prostate cancer cells, and inhibiting tumor growth *in vivo* (Chang et al., 2018). The inhibition of matrine on PC-3 cells is also associated with the regulation of Bim and p27 expression (Bai et al., 2017). However, studies have shown that matrine can inhibit tumor growth in DU145 xenograft model mice, but it is not effective in PC-3 xenograft model mice (Huang et al., 2017).

### Osteosarcoma

Matrine can induce apoptosis in human osteosarcoma MG-63 cells, but it also induces protective autophagy in MG-63 cells through extracellular signal-regulated kinase (ERK) signaling pathway, and inhibition of autophagy with chloroquine can enhance killing (Ma K. et al., 2016). Matrine also inhibits osteosarcoma cell proliferation and migration *via* the ERK/NF-kappaB signaling pathway (Li Y. et al., 2014). Matrine can also inhibit the growth of MNNG/HOS xenografts *in vivo* (Liang et al., 2012).

### Leukemia

Matrine can inhibit the expression of hsa-mir-106 b-3p and upregulate the expression of CDKN1A in human acute lymphoblastic leukemia (ALL) cell line CCRF-CEM, thereby blocking the cell cycle at G0/G1 phase and inducing apoptosis (Tetik et al., 2018). Matrine can increase the production of ROS in human acute lymphoblastic leukemia B cells, leading to mitochondrial swelling and mitochondrial membrane potential decline, thus inducing apoptosis (Aghvami et al., 2018). Matrine can also inhibit the proliferation of AML cells by inducing apoptosis and autophagy, and inhibit the phosphorylation of AKT, mTOR, and their downstream substrates p70S6K and eukaryotic translation initiation factor 4E binding protein 1(eIF4EBP1) (Wu J. et al., 2017). Matrine can also upregulate the expression of NKG2D ligand (NKG2DL) in leukemia cell lines and primary leukemia cells, and enhance the killing effect of NK and CIK cells on leukemia K562 cells (Zhang L. et al., 2015). The killing effect of Matrine on K562 cells is also related to interleukin-6 (IL-6)/JAK/STAT3 pathway (Ma L. et al., 2015).

### Other Cancers

Matrine can effectively inhibit the growth of glioblastoma multiforme (GBM) cells *in vitro* by inducing cell senescence, and downregulate the expression of insulin-like growth factor (IGF1), PI3K, and p-AKT. In an orthotopic xenograft model established by u251 and p3 cells transfected with luciferase, matrine inhibited tumor growth, and prolonged the overall survival of the animal model (Zhou W. et al., 2018). In addition, matrine can inhibit glioma cell metastasis and EMT, accompanied by inhibition of p38 MAPK and AKT signaling pathways (Wang et al., 2015b).

Matrine can induce mitochondrial-related endogenous apoptosis in retinoblastoma cells (Shao et al., 2014).

Matrine can regulate NF-κB to inhibit the migration and invasion of nasopharyngeal carcinoma cells (Sun and Xu, 2015).

Matrine can inhibit the proliferation of esophageal cancer Kyse-150 cells, induce ROS production and induce apoptosis. Matrine can destroy F-actin and nuclear structure. Morphological observation showed that the roughness and surface height of cell membrane increased with the increase of drug concentration (Jiang et al., 2018). Wang et al. (2014) reported that matrine acted on Eca-109 cells, induced apoptosis by upregulating p53 and p21, and arrested cell cycle in G0/G1 phase.

Matrine can significantly inhibit the proliferation and migration of cervical cancer cells by inhibiting p38 signaling pathway and inducing apoptosis (Wu X. et al., 2017).

Cisplatin is one of the first-line drugs for the treatment of urothelial bladder cancer (UBC), but its side effects and drug resistance become the limitations of its application. When the ratio of matrine to cisplatin was 2,000:1, it could synergistically inhibit UBC cells. The combination of the two drugs can inhibit the proliferation, invasion and EMT of UBC cells, induce cell cycle arrest and apoptosis, which is related to the signal pathway of VEGF/PI3K/AKT (Liao et al., 2017).

Matrine can inhibit the proliferation of rhabdomyosarcoma cells by inhibiting ERK signaling pathway and induce apoptosis (Yan et al., 2017). In combination with cisplatin, matrine can downregulate the expression of X-linked IAP (XIAP) and induce the apoptosis of rhabdomyosarcoma RD cells (Li L. et al., 2016).

Matrine combined with CYC116 can inhibit the proliferation of multiple myeloma RPMI8226 cells and induce apoptosis through PI3K/AKT pathway (Zhou et al., 2015).

It has been reported that matrine can inhibit the expression of miR-19b-3p and then upregulate PTEN, inhibit the proliferation and invasion of human A375 and SK-MEL-2 melanoma cell lines, and induce apoptosis (Wei Y. et al., 2018). Matrine can also upregulate PTEN expression and induce apoptosis in M21 cells (Jin et al., 2013).

**Figure 1:**
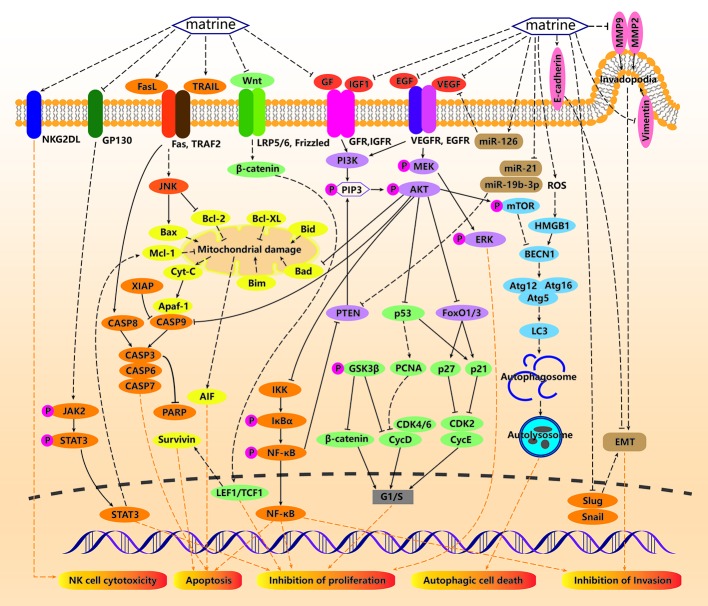
Anticancer mechanisms of matrine. For tumor cells, matrine can induce caspase-mediated exogenous apoptosis by activating Fas/Fas-L and TRAIL. Matrine can also induce mitochondrial damage by promoting the proapoptotic genes Bax, Bid, Bad, Bim, and downregulating the apoptosis-inhibiting genes Bcl-2, Mcl-1, Bcl-XL, and release Cyt-C and AIF to promote endogenous Apoptosis. Matrine can inhibit tumor cell proliferation through the GP130/JAK/STAT pathway, and can also induce apoptosis and inhibit proliferation by downregulating the expression of survivin through wnt/β-catenin and LEF1/TCF1. Matrine can inhibit insulin-like growth factor (IGF1) and GF and then affect the expression of phosphatidylinositol 3-kinase (PI3k)/AKT, nuclear factor κB (NF-κB) signaling pathway, and p53, thereby promoting tumor cell apoptosis, inhibiting proliferation and invasion. Matrine can also induce autophagy through PI3K/AKT/mTOR signaling pathway, causing autophagy related cell death and inhibiting the expression of EGF and vascular endothelial growth factor (VEGF). Matrine can also upregulate E-cadherin, downregulate MMP2, MMP9, and vimentin to inhibit invadopodia, slug, and snail, so as to inhibit epithelial-mesenchymal transition (EMT) and prevent tumor cell invasion. In addition, matrine can also promote the expression of NKG2DL in tumor cells to promote the recognition and killing of NK cells to tumor cells.

Antitumor related studies of matrine are summarized in **Table 1**, and the mechanisms of actions are summarized in **Figure 1**.

**Table 1:** Antitumor studies of matrine.

Diseases	Models	Effects	Mechanisms	References
Lung cancer	A549 cells	Apoptosis↑, proliferation↓, cell cycle arrest	miR-126↑, VEGF↓	(An et al., 2016)
		Apoptosis↑, proliferation↓, migration↓	p53↑, p21↑, PCNA↓, eIF4E↓	(Lu et al., 2017)
	A549, 95D cells	Apoptosis↑, proliferation↓	cIAP↓, p-AKT↓	(Niu et al., 2014)
	A549, NCI-H358 cells	ROS generation↑, apoptosis↑, proliferation↓	Cleaved caspase3↑, cleaved PARP↑, bcl-2↓, bad↑, p-p38↑	(Tan et al., 2013)
	A549, H1299 cells	Apoptosis↑, proliferation↓, migration↓	p-AKT↓, p-GSK3β↓	(Xie et al., 2018)
	LA795 cells; LA795 tumor bearing BALB/c mice	Proliferation↓, migration↓, tumor volume↓	TMEM16A↓	(Guo et al., 2018a)
	A549, H1299 cells	Proliferation↓, migration↓, EMT↓	PAX2↓, N-cadherin↓, E-cadherin↑, MMP2↓, MMP9↓	(Yang J. et al., 2017)
	A549, H460 cells	Apoptosis↑, proliferation↓	p-GSK3β↓, p-β-catenin↓, survivin↓, caspase3↑, caspase9↑	(Wang et al., 2015a)
Lung cancer (Matrine&afatinib)	H1975 cells; H1975 tumor bearing male BALB/c nude mice	Apoptosis↑, proliferation↓, tumor volume↓	p-JAK1↓, p-STAT3↓, IL6↓	(Chen et al., 2017)
Breast cancer	MCF-7, BT-474, MDA-MB-231 cells	Proliferation↓	IKKβ↓	(Shao et al., 2013)
	MCF-7 cells	Apoptosis↑, proliferation↓,	GRP78↑, eIF2α↑, CHOP↑, cyto-cyt-C↑, hexokinase II↓	(Xiao et al., 2017)
	4T1, MCF-7 cells; 4T1 tumor bearing BALB/c mice	Apoptosis↑, proliferation↓, tumor volume↓	Cleaved caspase9↑, cleaved caspase3↑, cyt-C↑, VEGF↓, wnt1↓, β-catenin↓, cyclin D1↓, c-Myc↓	(Xiao et al., 2018)
	MCF-7/ADR cells	Apoptosis↑, proliferation↓, intracellular concentration of ADR↑	p-gp↓, MRP1↓, p-AKT↓, bcl-2↓, PTEN↑, bax↑, cleaved caspase-3↑	(Zhou B. G. et al., 2018)
Liver cancer	HepG2, Huh7 cells	Apoptosis↑, viability↓, migration↓,proliferation↓, mitochondrial fission↑, cellular oxidative stress↑	Cleved caspase3↑, PARP↑, cadherin↓, vimentin↓, cyclin D1↓, CDK4↓, ROS↑, GSH↓,SOD↓, mito-Cyt C↓, Cyto-Cyt C↑,bax↑, caspase9↑, bad↑, bcl-2↓, c-IAP↓, mst1↑, p-JNK↑	(Cao et al., 2019)
	HepG2 cells	Apoptosis↑, proliferation↓, migration↓, mitophagy↓	Cleaved caspase3↑, PARP↑, cadherin↓, vimentin↓, cyclin D1↓, CDK4↓, mito-Cyt C↓, cyto-Cyt C↑, bax↑, caspase9↑, bcl-2↓, CIII-core2↓, CII-30↓, CIV-II↓, LC3-II↓, Atg5↓, vps34↓, PINK1↓, PARKIN↓	(Wei R. et al., 2018)
	HepG2 cells; HepG2 tumor bearing female BALB/c nude mice	Apoptosis↑, proliferation↓, tumor volume↓	Mito-cyt-C↓, cyto-cyt-C↑, HSP60↑, fas↑, fasL↑, mito-AIF↓, cyto-AIF↑, nuc-AIF↑	(Zhou et al., 2014)
	HepG2 cells	Apoptosis↑, proliferation↓, autophagy↑	Bax↑, beclin1↑	(Zhang et al., 2010)
	MHCC97L, Huh-7 cells; MHCC97L tumor bearing male BALB/c nude mice	Apoptosis↑, autophagy↑, proliferation↓,	Cleaved caspase3↑, cleaved caspase9↑, cleaved PARP↑, p62↓, LC3II↑, beclin1↑, PI3KC3↑, p-JNK↑, bcl-XL↑, bax↑, bak↑	(Yang and Yao, 2015)
	HepG2, SMMC7721 cells	Apoptosis↑, proliferation↓, autophagy↑	LC3II↑, p62↓, p-AKT↓, p53↓, p-ACC↑, CASP1↑, IFI27↑, IFITM1↑	(Xie et al., 2015)
	SMMC-7721-sphere cells	Proliferation↓	CAR↑, E-cadherin↑, laminin↑, fibronectin↑	(Wang H. et al., 2018)
	Huh-7 cells	Proliferation↓, migration↓, EMT↓	Cadherin↓, vimentin↓, Slug↓,Snail↓, MMP2↓, MMP9↓, PTEN↑, p-AKT↓	(Wang Z. et al., 2018)
Liver cancer (Matrine&resveratrol)	HepG2 cells	Apoptosis↑, proliferation↓, ROS generation↑	Survivin↓, PARP↑, bax↑, bcl-2↓	(Ou et al., 2014)
Liver cancer (Matrine&sorafenib)	HepG2, Hep3B cells	Apoptosis↑, proliferation↓	Cleaved caspase3↑, cleaved PARP↑, PTEN↑, miR-21↓	(Lin et al., 2014)
Cholangiocarcinoma	Mz-ChA-1, QBC939 cells	Necrosis↑, proliferation↓, ROS generation↑	RIP3↑	(Xu et al., 2017)
	Mz-ChA-1, KMCH-1 cells	Apoptosis↑, proliferation↓	Mito-cyto-C↓, cyto-cyto-C↑, caspase9↑, caspase3↑, p-JAK2↓, p-STAT3↓, Mcl-1↓	(Yang et al., 2015)
Gallbladder carcinoma	GBC-SD cells	Apoptosis↑, proliferation↓, cell cycle arrest	cleaved caspase3↑, bax↑, bcl-2↓, cyclin E↓	(Zhang et al., 2012)
Pancreatic cancer	Panc-1 cells	Apoptosis↑, proliferation↓, migration↓, EMT↓, cell cycle arrest, ROS generation↑	MMP-9↓, MMP-2↓, E-cadherin↓, N-cadherin↓, vimentin↓, p-IκBα↓, p-p65↓,	(Huang and Xin, 2018)
	MIAPACA2, 8988T cells; 8988T tumor bearing female SCID mice	Proliferation↓, autophagic degradation↓, tumor volume↓	p-STAT3↓, p62↑	(Cho et al., 2018)
	HPAC, Capan-1 cells	Proliferation↓, migration↓	MTI-MMP↓, MMP-2↓, MMP-9↓, Wnt↓, β-catenin↓	(Ma Y. et al., 2015)
	BxPC-3, Panc-1 cells; BxPC-3 tumor bearing male nude BALB/c mice	Apoptosis↑, proliferation↓, tumor volume↓	PCNA↓, cleaved caspase3↑, cleaved caspase9↑, cleaved caspase8↑, bax↑, bcl-2↓, fas↑	(Liu T. et al., 2010)
gastric cancer	SGC7901 cells	Proliferation↓, migration↓	p-ERK↓, p-AKT↓, uPA↓	(Peng et al., 2016)
	SGC-7901 cells	Proliferation↓	Regulating cell cycle, MAPK signaling pathway related miRNAs	(Li H. et al., 2014)
	BGC823 cells	Proliferation↓, migration↓	p-VASP↓, VASP↓	(Zhang et al., 2013)
	SGC7901, BGC823 cells	Autophagy induction↑, autophagic degradation↓	LC3-II↑, p62↑, procathepsin↑	(Wang et al., 2013)
	SGC7901 cells	Apoptosis↑, proliferation↓, autophagy↑	p-AKT↑, p-mTOR↑, p-P70S6K↑	(Li et al., 2013)
Colon cancer	LS174T, Caco-2, SW1116, RKO cells; LS174T tumor bearing BALB/c male nude mice	Apoptosis↑, proliferation↓, cell cycle arrest, tumor volume↓, tumor weight↓	Bcl-2↓, bax↑, cleaved caspase3↓	(Gu et al., 2018)
	1,2-dimethylhydrazine dihydrochloride treated male WISTAR rats	Tumor volume↓	HMGB1↓, IL-6↓, TNF-α↓	(Fan et al., 2018)
	LoVo cells	Apoptosis↑, proliferation↓	Cyclin D1↓, p27↑, p21↑, cleaved caspase9↑, bax↑, bcl-2↓, p-AKT↓, p-GSK3β↓	(Zhang et al., 2014)
	HT29 cells	apoptosis↑, proliferation↓, cell cycle arrest	cleaved caspase3↑, cleaved caspase9↑, bax↑, bcl-2↓, mito-cyt-C↓, cyto-cyt-C↑	(Chang et al., 2013)
Prostate cancer	DU145, PC-3 cells	Apoptosis↑, proliferation↓, migration↓	FOXO1α↓, FOXO3α↓, FOXO4↓, FOXO6↓, PI3K↓	(Li et al., 2018)
	DU145, PC3 cells	Apoptosis↑, proliferation↓, migration↓	Gadd45b↑	(Huang et al., 2018)
	DU145, PC3 cells; DU145 tumor bearing male BALB/c nude mice	Apoptosis↑, proliferation↓, migration↓, cell cycle arrest, EMT↓	E-cadherin↑, N-cadherin↓, vimentin↓, p-eIF2α↑, ATF4↑, CHOP↑, c-myc↓, bcl-2 ↓, bak↑, cleaved PARP↑	(Chang et al., 2018)
	DU145, PC-3 cells; DU145, PC-3 tumor bearing male BALB/c nude mice	Proliferation↓, migration↓, tumor volume in DU145 tumor bearing mice↓	p-MMP2↓, p-MMP9↓, p-p65↓	(Huang et al., 2017)
	PC-3 cells; Prostate epithelial cells RWPE1	Apoptosis↑, proliferation↓, cell cycle arrest	p27↑, CDK4↓, CDK2↓, bax↑, bim↑, bcl-2↓, p-AKT↓, p-FOXO3α↓	(Bai et al., 2017)
	DU145, PC-3 cells	Apoptosis↑, proliferation↓, cell cycle arrest	p-p65↓, p-IKKα/β↓, p-IκBα↓	(Li Q. et al., 2016)
Osteosarcoma	MG-63 cells	Apoptosis↑, proliferation↓, autophagy↑	p-ERK↑, LC3-II↑, bax↑	(Ma K. et al., 2016)
	SaOS-2, U2OS, MG-63 cells; U2OS tumor bearing male BALB/c nude mice	Proliferation↓, migration↓,	MMP-2↓, MMP-9↓, p65↓, p50↓, IκB-β↓, p-ERK↓	(Li Y. et al., 2014)
	MG-63, U-2OS, Saos-2, MNNG/HOS cells; MNNG/HOS tumor bearing female BALB/c mice	Apoptosis↑, proliferation↓, tumor volume↓	Cleaved caspase3↑, cleaved caspase9↑, cleaved caspase8↑, fas↑, fasL↑, bax↑, bcl-2↓	(Liang et al., 2012)
Leukemia	CCRF-CEM cells	Cell cycle arrest, apoptosis↑, proliferation↓	Hsa-mir-106b-3p↓, CDKN1A↑,	(Tetik et al., 2018)
	human ALL B-lymphocytes	Apoptosis↑, proliferation↓	Bax↑, bcl-2↓	(Aghvami et al., 2018)
	HL-60, THP-1, C1498 cells; C1498 tumor bearing C57BL/6 mice	Apoptosis↑, proliferation↓, autophagy↑, cell cycle arrest, spleen weight↓, survival↑	p62↓, LC3-II↑, PARP↑, cleaved caspase3↑, p-AKT↓,p-mTOR↓	(Wu J. et al., 2017)
	K562, OUN-1, HL-60, U937, K562/AO2 cells	NK and CIK cytotoxicity↑	NKG2DL↑, IL-6, IL-1, IL-2, IL-4, IL-5, GRO and TNF-α↓, CD158a ↓,CD158b↓	(Zhang L. et al., 2015)
	K562 cells	Apoptosis↑, proliferation↓, cell cycle arrest	Bcl-XL↓, cyclin D↓, c-myc↓, p-JAK2↓, p-STAT3↓, IL-6↓	(Ma L. et al., 2015)
Glioma	human glioma cell lines (U251, TCHu 58, U87MG, TCHu138); GFP- luciferase- stable U251 and P3 glioma cells bearing athymic mice	Proliferation↓, cell cycle arrest, induce cellular senescence, tumor growth↓, animal model survival↑	IGF1↓, PI3K↓, p-AKT↓	(Zhou W. et al., 2018)
	U251MG, U87MG cells	Proliferation↓, migration↓, EMT↓	E-cadherin↑, N-cadherin↓, p-p38↓, p-AKT↓,	(Wang et al., 2015b)
Retinoblastoma	SO-Rb50 cells	Apoptosis↑, proliferation↓	Apaf-1↑, cleaved caspase3↑, cleaved caspase9↑, cleaved caspase7↑, bax↑, bcl-2↓	(Shao et al., 2014)
Nasopharyngeal carcinoma	NPC-039, CNE-2Z cells; NPC-039 tumor bearing BALB/c nude mice	Proliferation↓, migration↓, tumor volume↓	MMP-2↓, MMP-9↓, p50↓, p65↓	(Sun and Xu, 2015)
Esophageal cancer	Kyse-150 cells	Apoptosis↑, proliferation↓, migration↓, ROS generation↑	Bax↑, caspase3↑, caspase8↑, caspase9↑, cleaved caspase8↑, bcl-2↓	(Jiang et al., 2018)
	Eca-109 cells; Eca-109 tumor bearing male nude BALB/c mice	Apoptosis↑, proliferation↓, cell cycle arrest, tumor volume↓	P53↑, p21↑, bid↑, bcl-2↓	(Wang et al., 2014)
Cervical cancer	Hela, C33A cells; Hela tumor bearing BALB/c athymic nude mice	Apoptosis↑, proliferation↓, migration↓, tumor volume↓	MMP2↓, MMP9↓, p38↓, p-AKT↓, p65↓	(Wu X. et al., 2017)
urothelial bladder cancer (Matrine&Cisplatin)	EJ, T24, BIU, 5637 cells	Apoptosis↑, proliferation↓, migration↓, EMT↓, ROS generation↑, cell cycle arrest	E-cadherin↑, β-catenin↑, fibronectin↓, vimentin↓, VEGFR2↓, VEGF↓, cleaved caspase3↑, bcl-2↓	(Liao et al., 2017)
Rhabdomyosarcoma	RD cells	Apoptosis↑, proliferation↓, migration↓	p-MEK1↓, p-ERK1/2↓, bcl-2↓, bax↑	(Yan et al., 2017)
Rhabdomyosarcoma (Matrine&cisplatin)	RD cells	Apoptosis↑, proliferation↓	XIAP↓	(Li L. et al., 2016)
multiple myeloma (Matrine&CYC116)	RPMI8226 cells	Apoptosis↑, proliferation↓	Cleaved caspase9↑, cleaved caspase3↑,cleaved PARP↑, bax↑, mcl-1↓, bcl-2↓, PI3K↓, p-AKT↓, NF-κB↓	(Zhou et al., 2015)
Melanoma	A375, SK-MEL-2 cells	Apoptosis↑, proliferation↓, migration↓	miR-19b-3p↓, PTEN↑	(Wei Y. et al., 2018)
	M21 cells	Apoptosis↑,proliferation↓, cell cycle arrest	p21↑, cyclinD1↓, bax↑, bcl-2↓, PTEN↑, p-PI3K↓	(Jin et al., 2013)

## Nonanticancer Activities

Matrine has therapeutic effects on Alzheimer's syndrome, encephalomyelitis, asthma, myocardial ischemia, rheumatoid arthritis (RA), and osteoporosis *in vitro* and *in vivo*. The spectrum of treatable diseases extends to many systems, such as nervous system, circulatory system, immune system and so on. Its mechanism is mainly to inhibit inflammation, reduce oxidative stress, regulate autophagy and apoptosis, etc. The antidisease effects and mechanisms of matrine are discussed in human body system and disease subsection below.

### Neurological Diseases

#### Alzheimer's Syndrome

It is estimated that 24 million people worldwide suffer from dementia, most of whom are thought to have Alzheimer's disease (AD). Therefore, AD is a major public health problem and a recognized research focus. Innovative therapies are urgently needed to cure or alleviate the disease (Ballard et al., 2011). Matrine has the potential to treat Alzheimer's syndrome. Matrine can inhibit the cytotoxicity induced by Aβ42, inhibit the Aβ/RAGE signaling pathway *in vitro*. Matrine reduces the deposition of proinflammatory cytokines and Aβ in AD transgenic mice and reduces memory deficit (Cui et al., 2017). It has been reported that matrine can reverse the changes of Th17/Treg cytokines induced by Aβ42 injection in AD rats, downregulate the expression of retinoid-related orphan receptor γt (RORγt), upregulate the expression of fork head box p3 (Foxp3), a specific transcription factor of Th17 cells, improve the learning and memory abilities of AD rats, and alleviate the cognitive impairment of AD rats (Zhang Y. et al., 2015).

#### Cerebral Ischemia

Matrine can alleviate cerebral ischemic injury, reduce the level of malondialdehyde (MDA), upregulate the expression of superoxide dismutase (SOD), glutathione peroxidase (GSH-px), catalase (CAT), and inhibit the apoptosis of ischemic neurons (Zhao et al., 2015).

#### Spinal Cord Injury/Encephalomyelitis

Matrine can promote axon growth and functional recovery in spinal cord injury (SCI) mice. Through drug affinity response target stability (DARTS) system screening, Matrine can directly bind heat shock protein 90 (HSP90), through neutralization. Specific blockade of anti-HSP90 by antibody can inhibit the growth of axons induced by matrine, suggesting that the improvement of SCI by matrine depends on the regulation of HSP90 (Tanabe et al., 2018). Matrine can also upregulate the expression of protein lipid protein, increase the number of mature oligodendrocytes and promote the formation of axonal myelin sheath in mice with autoimmune encephalomyelitis, which is related to PI3K/AKT/mTOR signaling pathway (Liu S. Q. et al., 2017). Matrine acts on experimental autoimmune encephalomyelitis (EAE) rats, which can upregulate the level of NGF and its receptor TrkA, inhibit the apoptosis of oligodendrocyte (OLG), and delay the course of disease (Zhu et al., 2016). In addition, this effect is also related to the downregulation of IL-33/ST2 expression in spinal cord of EAE rats (Zhao et al., 2016).

### Respiratory Diseases

#### Asthma

Matrine inhibits NF-κB signaling in airway epithelial cells and asthmatic mice, downregulates the expression of cytokine signaling 3 (SOCS3), and inhibits airway inflammation (Sun et al., 2016). Matrine can significantly reduce airway hyperresponsiveness (AHR) in asthmatic mice, and inhibit goblet cell hyperplasia, eosinophil infiltration and inflammatory response in lung tissue of asthmatic mice. Matrine also reduced the levels of Th2 cytokines and chemokines in bronchoalveolar lavage fluid and inhibited the production of OVA-IgE in serum. In addition, matrine treatment of activated BEAS-2B cells reduces the production of proinflammatory cytokines and eosinophil chemokines, as well as inhibits intercellular cell adhesion molecule (ICAM-1) expression and thus inhibits the adhesion of eosinophils and inflammatory BEAS-2B cells *in vitro*. Matrine can improve allergic asthma in mice and therefore has potential therapeutic potential (Huang et al., 2014).

#### Lung Injury

Matrine protects LPS-induced acute lung injury by inhibiting inflammatory responses, which may involve inhibition of ROS and tissue oxidative stress (Zhang et al., 2011).

### Circulatory Diseases

#### Cardiac Fibrosis

Cardiac fibrosis is one of the pathological features of diabetic cardiomyopathy (DBCM). Matrine can block transforming growth factor β1/receptor-regulated Smad (TGFβ1/RSMAD) signal transduction, inhibit collagen production and deposition in cardiac tissue, and alleviate high glucose-induced left ventricle. Impaired function and cardiac compliance (Zhang et al., 2018c). High glucose incubation induced activating transcription factor 6 (ATF6) signaling activation in CFS cultured *in vitro*, thereby increasing ECM synthesis. Matrine can inhibit ATF6, reduce myocardial fibrosis, and improve left ventricular function (Liu et al., 2017b).

#### Myocardial Ischemia

Myocardial ischemia is an important pathological process of coronary artery disease and has an important impact on cardiovascular outcomes (Rezende et al., 2019). Control of myocardial ischemia plays a very important role in coronary artery disease. Zhao et al. reported that matrine can alleviate apoptosis of cardiac microvascular endothelial cells (CMECs) induced by ischemia/reperfusion, which is related to JAK2/STAT3 signaling pathway (Zhao et al., 2018). Guo et al. (2018b) reported that matrine alleviated myocardial ischemia/reperfusion injury in rats by activating JAK2/STAT3 pathway, upregulating the expression of HSP70 and inhibiting myocardial apoptosis.

#### Diabetic Cardiomyopathy

Excessive ROS production in DBCM activates TLR-4/MyD-88 signaling, leading to cardiomyocyte apoptosis, while matrine preconditioning improves cardiac function by inhibiting ROS/TLR-4 signaling pathway (Liu et al., 2015).

#### Cardiotoxicity

Matrine has antioxidant properties and can alleviate isoproterenol-induced acute cardiotoxicity in rats (Li et al., 2010).

#### Heart Failure

Matrine inhibits cardiomyocyte apoptosis through the β3-AR pathway and improves cardiac function in rats with heart failure (Yu et al., 2014).

#### Vascular Injury

Matrine has the potential to treat vascular injury induced by high-fat diet. Matrine can alleviate abnormal lipid metabolism and inflammation in mice fed with high-fat diet, and significantly reduce oxidized low-density lipoprotein (ox-LDL) induced human umbilical vein endothelial cells (HUVECs). Other lial cells, HUVECs) dysfunction, alleviate the reduction of nitric oxide release, reduce the production of ROS, increase the expression of phosphorylated AKT-Ser473 and endothelial nitric oxide synthase-Ser1177 (eNOS-Ser1177). It can also downregulate the expression of eNOS-Thr495, a negative regulator of eNOS controlled by protein kinase C α (PKCα). Computational virtual docking analysis (AutoDock Vina software) and biochemical analysis showed that matrine affected eNOS/NO by inhibiting PKCα, and the protective effect of matrine could be eliminated by using PKCα and PI3K inhibitors (Zhang et al., 2019). Liu et al. (2018) reported that matrine can reduce AGEs-mediated Notch signal activation in human coronary smooth muscle cells (HCSMC), downregulate the expression levels of nicd1, hes1, collagen I, collegen VIII, and collagen secretion in HCSMC, and block the precondition of atheromatous plaque formation.

A cause of diabetic angiopathy is a high level of advanced glycation end products in the blood. Matrine can alleviate the damage of advanced glycation end products to aortic endothelial cells by inhibiting the activation of nod-like receptor protein 3 (NLRP3) inflammatory body mediated by ROS (Zhang et al., 2018d). Liu et al. reported that advanced glycation end products can induce ROS to induce endothelial cell apoptosis, which can lead to diabetic vascular complications. Matrine restored phosphorylation of MKKK3/6 and p38 MAPK, nuclear translocation of nuclear factor-erythroid 2-related factor 2 (Nrf2), binding activity of antioxidant response elements and expression level, inhibited ROS production and endothelial cell apoptosis *in vitro* and *in vivo* (Liu et al., 2017a).

### Digestive Diseases

#### Liver Fibrosis

Hepatic fibrosis is a wound healing reaction characterized by the accumulation of extracellular matrix after various liver injuries, which leads to the deformation of normal liver structure and develops into cirrhosis and even hepatocellular carcinoma (Lin et al., 2018). Controlling liver fibrosis in time can prevent the transformation of malignant diseases. Mahzari et al. (2018) reported that in two models of liver fibrosis with abnormal glucose metabolism induced by high fructose diet (HFRU), high fat diet (HF) and low dose streptozotocin (STZ), matrine intervention can upregulate heat-shock protein 72 (HSP72) to inhibit liver fibrosis and improve blood sugar level. For carbon tetrachloride (CCl4)-treated hepatic stellate cell inflammation and fibrosis models, matrine can inhibit the production of MCP-1 and reduce the infiltration of Gr1(hi) monocytes in liver tissue, reducing liver inflammation and fibrosis (Shi et al., 2013).

#### Fatty Liver

Matrine can inhibit blood sugar and lipid abnormalities in mice fed with high-fat diet and alleviate liver steatosis. Compared with metformin, matrine neither inhibits mitochondrial respiration nor activates AMPK in liver. The regulation of matrine is related to the activation of HSP72 (Zeng et al., 2015).

#### Pancreatic Fibrosis

Matrine can alleviate rat pancreatic fibrosis induced by Trinitrobenzene sulfonic acid. Matrine reduces glandular hyperplasia, reduces mitochondrial swelling of acinar cells, and downregulates α-smooth muscle actin (α-SMA), TGF-β, and collagen In addition, Smad2, TβR1, and TβR2 were significantly downregulated in mRNA and protein levels (Liu et al., 2019).

#### Colitis

Matrine can alleviate the symptoms of spontaneous colitis in IL-10 deficient mice and reduce the expression levels of IL-12/23p40, interferon-γ (IFN-γ), IL-17 in colon tissues (Wu et al., 2016).

### Urinary System Disease

#### Adriamycin-Induced Nephropathy

Matrine can alleviate nephropathy caused by doxorubicin treatment *via* the Foxp3/RORγt pathway [111].

### Immune System Disease

#### Rheumatoid Arthritis

Overproliferation and intrinsic resistance to apoptosis of fibroblast-like synoviocytes (FLS) are important pathogenesis of RA. Matrine can reduce arthritis index (AI) by acting on collagen-induced arthritis (CIA) model in rats. *In vitro*, matrine inhibits the proliferation of FLS, induces cell cycle arrest of G0/G1 cells, and inhibits the activation of JAK/STAT signaling pathway, thereby increasing the apoptotic rate *in vitro* (Yang Y. et al., 2017). Rat rheumatoid arthritis model is characterized by Th1/Th2 imbalance. Matrine reduces the level of Th1 cytokines, such as IFN-γ, tumor necrosis factor (TNF-α), IL-1β, by regulating the NF-κB signaling pathway, and increases Th2 cytokines (IL-4 and IL-10) to balance the Th1/Th2 axis (Niu et al., 2017).

### Osteopathy

#### Osteoporosis

The imbalance between the osteogenic effects of osteoblasts and the osteoclasts of osteoclasts is one of the pathogenesis of postmenopausal osteoporosis. Secretion of estrogen causes an increase in the level of proinflammatory cytokines. Inflammation-induced osteoclast hyperactivity plays a crucial role in the imbalance. Matrine can inhibit osteoclastogenesis, inhibit inflammation and alleviate osteoporosis by regulating the NF-κB/AKT/MAPK pathway (Chen et al., 2017b).

#### Chondropathy

Matrine can inhibit the activation of MAPK and NF-κB in human chondrocytes *in vitro* to inhibit IL-1β-induced MMP expression, thereby inhibiting MMP degradation of extracellular matrix and inhibiting chondrocyte apoptosis (Lu et al., 2015).

### Mental Disease

#### Anxiety and Depression Induced by Liver Injury

Matrine can alleviate neuro-inflammation and oxidative stress in the brain caused by acute liver injury, thus producing antianxiety and antidepression effects. CCl4 induces acute liver injury in mice. Matrine pretreatment can significantly improve anxiety and depression-like behavior, alleviate neuro-inflammation, downregulate the levels of proinflammatory factors TNF-α, IL-1β, and IL-6, and increase the levels of glutathione (GSH), catalase (CAT), and glutathione S-transferase in brain tissue of mice. The level of GST decreased the levels of MDA and nitrite in brain tissue, thus reducing the oxidative stress induced by CCl4. Matrine significantly reduced the contents of corticosterone, ammonia, glutamic oxalate transaminase, glutamic oxalate transaminase and creatinine, and significantly improved CCl4-induced liver morphological damage. Matrine treatment increased the levels of glial fibrillary acidic protein (GFAF) positive astrocytes, brain-derived neurotrophic factor (BDNF), and VEGF in the hippocampus of mice to promote neurogenesis and inhibit hippocampal neuronal apoptosis (Khan et al., 2019).

### Cancer-Associated Skeletal Muscle Atrophy

Cancer cachexia is a complex condition secondary to systemic progressive dysfunction and tissue atrophy secondary to cancer. Cancer cachexia is characterized by systemic inflammation, negative energy, and protein balance, generally with weight loss associated with skeletal muscle atrophy, and adipose tissue depletion (Argiles et al., 2010; Fearon et al., 2011). Matrine can increase muscle fiber size and muscle mass in a mouse model of CT26 colon cancer cachexia *in vivo*. At the same time, it relieves cachexia symptoms such as body and organ weight loss. *In vitro*, matrine also attenuated dexamethasone, TNF-α, and conditioned medium-induced c2c12 myotube atrophy and apoptosis. This process is associated with activation of the AKT/mTOR/Foxo3α signaling pathway. In addition, matrine downregulates the expression of the E3 ubiquitin ligases muscle-specific RING finger protein 1 (MuRF1) and muscle atrophy F-box protein (MAFbx) (Chen et al., 2019).

**Figure 2:**
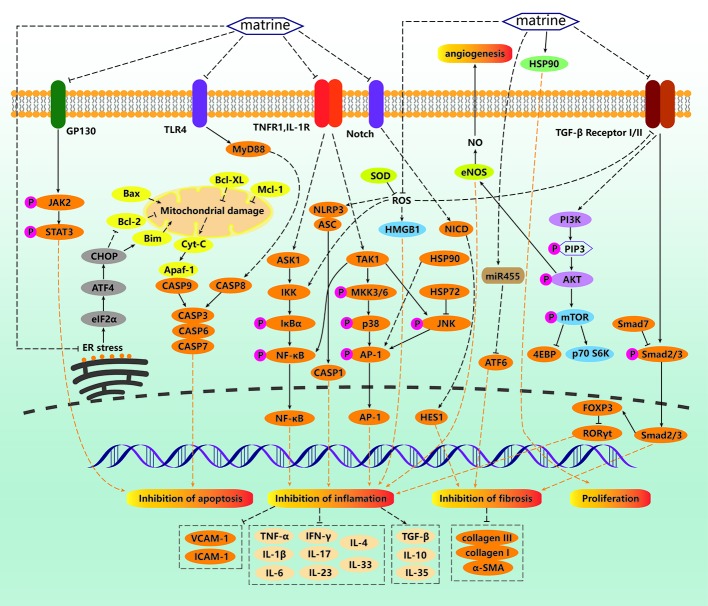
Non-anticancer mechanisms of matrine. For normal cells, matrine can promote cell survival under various stress environments. Under oxidative stress conditions, matrine can inhibit reactive oxygen species (ROS) production, thus inhibiting high mobility group protein box 1 (HMGB1), nod-like receptor protein 3 (NLRP3)/ASC/CASP1 pathway and nuclear factor κ B (NF-κB pathway)–mediated inflammation. Matrine can also inhibit tumor necrosis factor (TNF)-α and IL-1 induced NF-κB and TAK/JNK/AP1 pathway-mediated inflammation. In addition, TGFβ/Smad/FOXP3/RORγt is also a pathway for matrine to inhibit inflammation. Matrine can also block CASP8 mediated exogenous apoptosis by inhibiting TLR4/MyD88 pathway, eIF2α/ATF4/CHOP mediated mitochondrial damage, Cyt-C release and CASP9 mediated endogenous apoptosis by inhibiting ER stress. Matrine can also inhibit GP130/JAK/STAT pathway mediated apoptosis. TGFβ/Smad, NOTCH/NICD, and miR455/ATF6 mediated fibrosis can also be inhibited by matrine. In addition, matrine can promote cell proliferation by activating Hsp90.

Non-antitumor related studies of matrine are summarized in **Table 2**, and the mechanisms of actions are summarized in **Figure 2**.

**Table 2:** Non-anticancer studies of matrine.

Diseases	Models	Effects	Mechanisms	References
Alzheimer's disease	Aβ42 treated SH-SY5Y cells; APP/PS1 transgenic mice	Cell viability↑, inflamation↓	BACE1↓, NF-κB↓, TNF-α↓, IL-1β↓	(Cui et al., 2017)
	Aβ1-42 treated Sprague Dawley rats	Cognitive ability (water maze test) ↑, novel object recognition test↑	IL-17A↓, IL-23↓, TGF-β↑, IL-35↑, RORγt↓, foxp3↑	(Zhang L. et al., 2015)
Cerebral ischemia	MCAO mice	Brain infract volume↓, apoptosis↓,	Caspase3↓, bax↓, bcl-2↑, MDA↓, SOD↑, GSH-Px↑, CAT↑	(Zhao et al., 2015)
Spinal cord injury	Cortical neurons; female ddY mice	Motor dysfunction↓, density of 5-HT-positive tracts↑	HSP90↑	(Tanabe et al., 2018)
Autoimmune encephalomyelitis	EAE C57BL/6 mice model	OPC proliferation↑, oligodendrocyte numbers and PLP expression↑	p-PI3K↑, p-AKT↑, p-mTOR↑, p-p70S6K↑	(Liu S. Q. et al., 2017)
Multiple sclerosis	experimental autoimmune encephalomyelitis female WISTAR rats	OLG apoptosis↓	NGF↑, trkA↑	(Zhu et al., 2016)
	experimental autoimmune encephalomyelitis female WISTAR rats	Clinical score↓	IL-33↓, ST2↓	(Zhao et al., 2016)
Asthma	human bronchial epithelial cell line BEAS-2B and MLE-12 mouse lung epithelial cells; OVA treated female BALB/c mice	Asthmatic symptoms↓, inflamation↓	SOCS3↓, ICAM1↓, VCAM1↓, p-p65↓	(Sun et al., 2016)
	BEAS-2B cells; Ovalbumin(OVA) treated female BALB/c mice	Inflamation↓, airway hyperresponsiveness↓	IL-4↓, IL-5↓, IL-6↓, IL-13↓, TNF-α↓, IgE↓	(Huang et al., 2014)
Lung injury	LPS treated male BALB/c mice	Inflamation↓, survival↑	MPO↓, MDA↓, plasma TNF-α↓, IL-6↓, HMGB1↓, p-NF-κB↓	(Zhang et al., 2011)
Diabetic cardiomyopathy	HG medium cultured cardiac fibroblasts; streptozotocin treated Sprague Dawley rats	Fibrosis↓, left ventricular functions↑, cardiac compliance loss↓	TGF-β1↓, p-smad2↓, p-smad3↓, smad7↓, collagen I↓	(Zhang et al., 2018c)
	Streptozotocin treated male Sprague Dawley rats	Myocyte apoptosis↓, ROS generation↓	MDA↓, GPx↑, TLR4↓, MyD-88↓, cleaved-caspase8↓, cleaved-caspase3↓	(Liu et al., 2015)
Cardiac fibrosis	cardiac fibroblasts from 2-day-old Sprague Dawley rats; streptozotocin treated Sprague Dawley rats	Cardiac systolic/diastolic dysfunction↓, cardiac compliance↑	ATF6↓, miR455↑, calreticulin↓, fibronectin↓, collagen I↓	(Liu et al., 2017b)
Hypoxia/reoxygenation induced cardiac microvascular endothelial cells death	oxygen free anoxic solution treated rat cardiac microvascular endothelial cells (CMECs)	Apoptosis↓, tube formation ability↑	p-JAK2↑, p-STAT3↑, bcl-2↑, bax↓	(Zhao et al., 2018)
Myocardial ischemia/reperfusion (I/R) injury	neonatal rat cardiomyocytes; Male Sprague Dawley rats	Cell viability↑	Bax↓, bcl-2↑, caspase3↓, CK-MB↓, cTnI↓, p-JAK2↑, p-STAT3↑, HSP70↑	(Guo et al., 2018b)
Isoproterenol-induced acute cardiotoxicity	Isoproterenol treated male Sprague Dawley rats	Heart function↑, inflamation↓	SOD, catala, glutathione peroxidase↑, MDA↓	(Li et al., 2010)
Heart failure	Apply coronary artery ligation to establish rat heart failure model	Cardiac function↑, apoptosis↓	Cleaved caspase3↓, bax↓, bcl-2↑, β3AR↓, eNOS↓	(Yu et al., 2014)
Lipid metabolism disorders caused vascular endothelial injury	The HUVECs treated with ox-LDL; Male C57BL/6 mice were given high-fat diet for 12 weeks	Lipid metabolism↑, inflamation↓, thickness of vascular wall↓, ox-LDL-induced apoptosis↓	Serum TNF-α↓, IL-6↓, IL-10↑, p-AKT-Ser473↑, eNOS-Ser1177↑, eNOS-Thr495↓	(Zhang et al., 2019)
Atherosclerosis	AGEs treated HCSMCs	Contractile synthetic phenotypic conversion↓	DLL4↓, notch↑, collagen I↓, collegen VIII↓, NICD1↓, HES1↓,	(Liu et al., 2018)
Advanced glycation end products (AGEs) induced damage in the arterial endothelium;	AGEs treated human aortic endothelial cells	Cell viability↑, infamation↓, intracellular reactive oxygen species↓	NLRP3↓, ASC↓, cleaved caspase-1↓, IL-1β↓	(Zhang et al., 2018d)
Diabetic vascular complications	AGEs treated Sprague Dawley rats; Rat aortic endothelial cells	ROS generation↓, apoptosis↓	p-MKK3↑, p-MKK6↑, p-38↑, HO1↑, NQO1↑, nrf2↑	(Liu et al., 2017a)
Hepatosteatosis with glucose intolerance	high-fructose diet (HFru) induced hepatosteatosis and glucose intolerance from hepatic, and hepatosteatosis and hyperglycemia induced by high-fat (HF) diet in combination with low doses of streptozotocin (STZ); C57BL/6J mice	Body weight↓, epididymal fat weight↓, triglyceride↓	SREBP-1c↓, ChREBP↓, SCD-1↓, fas↓, eIF2α↓, CHOP↓, IRE1↓, HSP72↑	(Mahzari et al., 2018)
Liver fibrosis	CCl4 treated C57BL/6mice	Inflamation↓	MCP-1↓, number of CD45^+^ cells↓, number of Gr1^+^ cells↓	(Shi et al., 2013)
Hepatic steatosis	high-fat-fed C57BL/J6 mice	Glucose intolerance↓, hepatosteatosis↓, inflamation↓	TNF-α↓, IL-6↓,IL-1β↓,nSREBP-1↓, SCD-1↓, UCP2↑,HSP72↑,	(Zeng et al., 2015)
Pancreatic fibrosis	Sprague Dawley rats, 12.5 mL of 2% trinitrobenzene sulfonic acid-ethanol phosphate buffer solution containing 1 mL of 5% trinitrobenzene sulfonic acid and 1.5 mL of 10% ethanol phosphate buffer solution were injected in pumpbiliopancreatic duct with a micro-injection	Mitochondrial swelling of acinous cells↓, hyperplasia of glandules↓, fibrosis↓	α-SMA↓, TGF-β1↓, collagen I↓, smad2↓, TβR1↓, TβR2↓	(Liu et al., 2019)
Chronic colitis	IL-10 deficient mice	Inflamation↓	IFN-γ↓, IL-17↓,	(Wu et al., 2016)
Adriamycin-induced nephropathy	Adriamycin treated male Sprague Dawley rats	Renal function↑, inflamation↓	Foxp3↑, RORγt↓	(Xu et al., 2016)
Rheumatoid arthritis	Bovine type II collagen treated fibroblast-like synoviocytes; bovine type II collagen treated male Sprague Dawley rats	Apoptosis↑, proliferation↓, cell cycle arrest, arthritis index↓	p-JAK2↓, p-STAT1↓, p-STAT3↓, bax↑, bcl-2↓, caspase3↑	(Yang Y. et al., 2017)
	bovine type II collagen treated male Sprague Dawley rats; phorbol myristate acetage (PMA) and ionomycin treated BALB/c mice splenic CD3^+^ T lymphocytes	Inflamation↓,	p65↓,p-IκBα↓, IFN-γ↓, TNF-α↓, IL-1β↓, IL-4↑, IL-10↑	(Niu et al., 2017)
Osteoporosis	bone marrow monocytes, RAW264.7 cells; RANKL treated C57BL/6 mice	Osteoclastogenesis↓, inflamation↓	Serum TRAcp5b↓, TNF-α↓, IL-6↓, MMP9↓, NFATc1↓, TRAP↓, c-src↓, cathepsin K↓, p-ERK↓, p-JNK↓, p-p38↓, p-AKT↓	(Chen et al., 2017b)
	IL-1β treated human articular cartilage	Apoptosis↓, chondrocyte viability↑	p-p38↓, p-ERK↓, p-JNK↓, IκBα↑	(Lu et al., 2015)
Acute liver injury induced neuroinflammation and oxidative stress	CCl4 treated male BALB/c mice	Food intake↑, water intake↑, inflamation↓, open field test (OFT) ↑, elevated plus maze test (EPM) ↑, light-dark box test (LDB) ↑, forced swimming test (FST) ↑, and tail suspension test (TST) ↑, apoptosis↓	Hippocampus and prefrontal cortex TNF-α↓, IL-6↓, IL-1β↓; GSH↑, GST↑, CAT↑, NO↓, MDA↓, ammonia↓, corticosterone↓, GFAF↑, BDGF↑, VEGF↑, caspase-3↓	(Khan et al., 2019)
Cancer induced cachexia and muscle atrophy	CT26 tumor bearing BALB/c mice; TNF-alpha, dexamethasone, conditioned medium treated C2C12 myotubes	Muscle weight↑, C2C12 myoblast differentiation↑	MuRF1↓, MAFbx↓,p-AKT↑, p-mTOR↑, p-FOXO3α↑	(Chen et al., 2019)

## Discussion and Prospect

Cancer is one of the most serious diseases in the history of human health, for which the whole society bears a huge material and spiritual burden. In 2018, it is estimated that 18.1 million new cancer cases and 9.6 million cancer deaths will occur globally (Bray et al., 2018). With the development of cancer treatment methods, the overall survival rate of cancer has increased, but it is still not optimistic (Allemani et al., 2018). Currently, the main methods of cancer treatment are surgery, chemotherapy, radiotherapy, and targeted therapy. In recent years, immunotherapy represented by immuno-checkpoint inhibitors, chimeric antigen receptor-T (CAR-T) therapy and cancer vaccine has made tremendous progress (Yang, 2015). But immunotherapy is not applicable to all cancer patients (Beatty and Gladney, 2015). Although individual cancer vaccines have emerged to respond to individual mutations (Sahin and Tureci, 2018), the high cost of treatment makes it impossible to benefit most patients. In short, the treatment of cancer cannot meet the current situation.

Natural product therapy (NPT), as an alternative treatment for cancer, has attracted much attention. Many natural products have high potential for direct treatment of cancer (Newman and Cragg, 2016; Dutta et al., 2019), or have the effect of improving drug resistance and enhancing the efficacy of anticancer drugs. At present, the dosage forms are also constantly upgrading (Gerber et al., 2013; Watkins et al., 2015; Kashyap et al., 2019). Compared with targeted therapy and immunotherapy, natural products have great advantages in cost, which deserve further research and clinical promotion. Matrine is a natural product with a variety of activities and high conversion value.

Matrine can inhibit the proliferation of more than ten kinds of tumor cells, mostly by inducing apoptosis, blocking cell cycle and inhibiting cell migration. Matrine can also induce autophagy of tumor cells, such as hepatocellular carcinoma cells (Zhang et al., 2010; Xie et al., 2015; Yang and Yao, 2015), gastric cancer cells (Li et al., 2013; Wang et al., 2013), osteosarcoma cells (Ma K. et al., 2016), acute myeloid leukemia cells (Wu J. et al., 2017). In some tumors, such as hepatocellular carcinoma HepG2 cells (Xie et al., 2015), osteosarcoma MG-63 cells (Ma K. et al., 2016), it is protective autophagy. Matrine can be used in pancreatic cancer and gastric cancer. Inhibiting the protective effect of autophagy, blocking the degradation process of substrates and promoting apoptosis (Li et al., 2013; Wang et al., 2013; Cho et al., 2018). Autophagy is a biological process with multifaceted effects, which can promote cell survival and induce death (Jiang et al., 2019; Mirza-Aghazadeh-Attari et al., 2019). However, the nature of autophagy remains unclear in many studies, and needs to be further explored.

In addition, many derivatives of matrine also have antitumor, antifibrosis, and antiosteoporosis effects. Qian et al. reported that WM130, a matrine derivative, could inhibit the proliferation, invasion and migration of HCC cells by inhibiting EGFR/ERK/MMP-2 and PTEN/AKT signaling pathways and induce apoptosis of hepatocellular carcinoma cells (Qian et al., 2015). Matrine derivative WM-127 can induce cell cycle arrest and apoptosis of hepatocellular carcinoma HepG2, Hep3B, Huh7, LM3, SMMC-7721 by regulating Survivin/beta-catenin signaling pathway (Yin et al., 2018). Matrine derivatives (6aS, 10S, 11aR, 11bR, 11cS)-10-methylamino-dodecahydro-3a, 7a-diazabenzo (de) (MASM) can inhibit the proliferation, cell cycle and apoptosis of hepatocellular carcinoma cells through PI3K/AKT/mTOR and AKT/GSK3β/β-catenin signaling pathways, inhibit the growth of tumors and inhibit the dryness of tumor cells (Liu Y. et al., 2017). MASM can also inhibit ribosomal protein S5 (RPS5), and regulate PI3K/AKT, NF-κB, and MAPKS pathways to inhibit osteoclastogenesis. MASM has the potential to become a drug for osteoporosis (Chen et al., 2017a). Xu et al. reported that WM130, a matrine derivative, could inhibit apoptosis, ECM deposition, TGF-β/Smad and Ras/ERK pathways, HSC-T6 cell activation and hepatic fibrosis in rats (Xu et al., 2015).

Matrine can regulate noncoding RNA and then affect key molecules related to cancer progression, such as upregulation of miR-126 to inhibit VEGF (An et al., 2016), downregulation of miR-21 and miR-19b-3p, and alleviate the inhibition of PTEN (Lin et al., 2014; Wei Y. et al., 2018).

Matrine has strong antiinflammatory and antiapoptotic effects in nonneoplastic diseases, such as protecting normal cells in cell and animal models of AD (Cui et al., 2017), asthma (Sun et al., 2016), lung injury (Zhang et al., 2011), liver fibrosis (Shi et al., 2013), colitis (Wu et al., 2016). Matrine can also upregulate the expression of miR-455, thereby inhibiting fibrin synthesis and alleviating myocardial fibrosis (Liu et al., 2017b).

**Figure 3:**
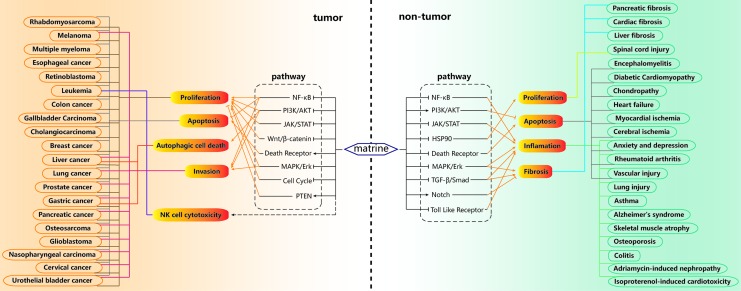
Summary of signal pathways and diseases related to the actions of matrine. For tumor cells, matrine can inhibit proliferation and invasion, promote apoptosis and autophagic cell death, and enhance the cytotoxicity of NK cells. These effects are related to the inhibition of nuclear factor κ B (NF-κB), phosphatidylinositol 3-kinase (PI3K)/AKT, Wnt/β-catenin, mitogen-activated protein kinase (MAPK)/extracellular signal-regulated kinase (ERK) and cell cycle pathways, and promotion of PTEN, death receptor pathways. For normal cells, matrine can promote proliferation, inhibit apoptosis, inflammation and fibrosis. These effects are related to matrine inhibiting NF-κB, JAK/STAT, Hsp90, MAPK/ERK, TGFβ/Smad, death receptor, toll like receptor pathways, promoting PI3K/AKT, NOTCH pathways.

It is worth noting that matrine can inhibit proliferation and induce apoptosis in cancer cells, while for normal cells in pathological environment, matrine can inhibit apoptosis and maintain growth and proliferation (The signaling pathways and diseases related to the actions of matrine are summarized in **Figure 3**). NF-κB is involved in the inflammatory response and immune response of the body, and can regulate cell apoptosis and stress response (Pires et al., 2018). Matrine can inhibit NF-κB to inhibit the proliferation, invasion and apoptosis of tumor cells. When normal cells such as nerve cells, tracheal epithelial cells and chondrocytes are under stress, matrine can inhibit NF-κB to inhibit apoptosis and inflammation to maintain the survival of normal cells (Zhang et al., 2011; Lu et al., 2015; Cui et al., 2017; Niu et al., 2017). JAK/STAT regulate the expression of a variety of proteins involved in induction or prevention of apoptosis, and has also become a paradigm for membrane-to-nucleus signaling and explains how a broad range of soluble factors, including cytokines and hormones, mediate their diverse functions (Villarino et al., 2015; Bousoik and Montazeri Aliabadi, 2018). Matrine inhibits JAK/STAT pathway to inhibit tumor cell proliferation and inflammation in normal cells (Ma L. et al., 2015; Yang et al., 2015; Guo et al., 2018b; Zhao et al., 2018). The MAPKs in mammals include JNK, p38 MAPK, and ERK. These enzymes are serine-threonine protein kinases that regulate various cellular activities including proliferation, differentiation, apoptosis or survival, inflammation, and innate immunity. The compromised MAPK signaling pathways contribute to the pathology of diverse human diseases (Kim and Choi, 2015; Sun et al., 2015). Matrine negatively regulates the MAPK/ERK pathway, thereby inhibiting tumor cell proliferation and suppressing the inflammatory response or fibrosis in normal cells (Lu et al., 2015; Wang et al., 2015b; Peng et al., 2016; Yan et al., 2017). PI3Ks are crucial coordinators of intracellular signaling in response to the extracellular stimulators. The serine/threonine kinase AKT is a master regulator of many diverse cellular functions, including survival, growth, metabolism, migration, and differentiation. The signaling axis formed by PI3K and AKT, as well as the vast range of downstream substrates is thus central to control of cell physiology in many different contexts and tissues (Noorolyai et al., 2019; Sugiyama et al., 2019). Matrine inhibits the PI3K/AKT pathway in tumor cells, while the reverse occurs in normal cells (Niu et al., 2014; Xie et al., 2015; Liu S. Q. et al., 2017; Wu X. et al., 2017; Chen et al., 2019; Zhang et al., 2019). Moreover, for tumor cells, matrine induces oxidative stress leading to endogenous apoptosis in cells, while in ischemic disease models, matrine inhibits oxidative stress and thereby inhibits apoptosis of nerve cells and cardiac muscle cells. In different application environments, matrine has contradictory effects. In addition, natural compounds such as resveratrol (Ko et al., 2017; Xia et al., 2017), baicalein (Liu et al., 2016; Sowndhararajan et al., 2017), and quercetin (Li Y. et al., 2016; Massi et al., 2017) also have similar killing and protecting effects. However, there is still no reasonable and accepted explanation for this dualistic effect. We speculate that this phenomenon may be related to different cell properties. We can find tumor cells that cannot be inhibited by matrine and normal cells that cannot be protected in a stressful environment or even directly inhibited, and make a histological analysis of these cells, which may be helpful to find the mechanism of this phenomenon.

The underlying mechanism of matrine's selective killing of cells remains to be explored. The role of matrine in autophagy induction or inhibition needs to be further determined. In addition, the regulation of noncoding RNA by matrine may be an important way for matrine to play its role. Whether there are transcriptome-related intrinsic regulatory roles in all disease models needs to be further explored.

Last but not least, the pharmacokinetics of matrine in different modes of administration need to be further improved. The most common way to take matrine is oral administration. A liquid chromatography/tandem mass spectrometry (LC/MS/MS) developed method facilitated a clinical pharmacokinetic study after oral administration of a single dose of matrine soft gelatin capsules (100, 200 and 400mg) in a three-period crossover design. Dose-related linear trends were observed for the AUC_0-t_ and the C_max_ of matrine. The t_1/2_ and the T_max_ of matrine were independent of the administered doses (Zhang et al., 2009). Another study explored the pharmacokinetics of matrine through intravenous injection or transdermal administration in rat liver, blood, skin and other organs and tissues, and found that transdermal administration is also a promising way (Tang et al., 2017). At present, there is no pharmacokinetic study of matrine for specific diseases.

In summary, matrine has a wide range of pharmacological effects and high development value, and further mechanism research needs to be carried out.

## Author Contributions

HZ and LC prepared the manuscript. HZ, XS, and QY edited the tables and figures. LW and CG revised the manuscript. All authors approved the final manuscript.

## Funding

This work was supported by the National Natural Science Foundation of China (Nos. 81873042, 81872494, and 81803633).

## Conflict of Interest

The authors declare that the research was conducted in the absence of any commercial or financial relationships that could be construed as a potential conflict of interest.
